# Control of Passion Fruit Fungal Diseases Using Essential Oils Extracted from Rosemary* (Rosmarinus officinalis)* and Eucalyptus* (Eucalyptus agglomerata)* in Egerton University Main Campus Njoro, Kenya

**DOI:** 10.1155/2017/2814581

**Published:** 2017-03-28

**Authors:** Paul Njenga Waithaka, Eliud Mugu Gathuru, Benson Muriuki Githaiga, Salome Nduta Kimani

**Affiliations:** ^1^School of Biological Sciences, University of Nairobi, P.O. Box 30197-00100, Nairobi, Kenya; ^2^Department of Biological Sciences, Egerton University, P.O. Box 536, Njoro, Kenya

## Abstract

Growth of fruits which form an important part of human diet has been jeopardized by the many fungal diseases that are present today. This study was conceived to isolate the most common fungal pathogens in passion fruits. Fungi were isolated using potato dextrose agar in addition to characterization using morphological, cultural, and biochemical means. Extraction of essential oils from rosemary (*Rosmarinus officinalis*) and eucalyptus (*Eucalyptus agglomerata*) was done. Before carrying the sensitivity test of essential oils to the fungal isolates, constituents of the essential oils were determined. The most common fungal pathogens isolated from passion fruits were* Alternaria *spp. (45%),* Fusarium *spp. (22%),* Colletotrichum *spp. (17%), and* Penicillium *spp. (16%). There was a relationship between heating time and yield of essential oils in rosemary (*r* = 0.99) and eucalyptus (*r* = 0.99). Conversely, there was no significant difference in the amount of essential oils produced by rosemary and eucalyptus (*P* = 0.08). Furthermore, there was a significant difference in growth inhibition of the fungal pathogens between essential oils from rosemary and eucalyptus (*P* = 0.000438). Fungal pathogens isolated from passion fruits can be controlled using essential oils from rosemary and eucalyptus. The oils need to be produced in large scale.

## 1. Introduction

Fruits are important domestic, industrial, and commercial foods. They provide vitamins and essential mineral salts which boost the body's immune system [1]. Passion fruits are hosts of many fungal pathogens which provoke severe losses of agricultural and horticultural crops every year (Parveen et al., 2016). Although passion fruits have wide distribution in nature, their growth is limited by the proliferation of fungal pathogens that threaten their growth. It is estimated that 20–25% of the harvested passion fruits are deteriorated by fungal pathogens during postharvest handling [1].

The postharvest losses due to fungal pathogens in passion fruits are more severe in developing countries due to poor storage and transportation facilities [2]. Passion fruits infections by fungi may appear during the growth period, harvesting, handling, transportation, and postharvest stockpile and marketing conditions, or after procurement by the consumer [3]. Due to their high levels of nutrients element and sugars coupled with low pH, passion fruits have an exceptionally desirable microclimate for proliferation of fungal pathogens [4]. Fungi are considered as an essential postharvest losses agent of different varieties of passion fruits, based on cultivar, season, and production area amid other factors [5].

Fungi are the most crucial and common pathogens and the main cause of crop diseases due to their ability to propagate by spores. Since the spores are plenty in air, fungal pathogens infect a wide range of fruits and vegetables during storage and transportation [6]. In addition, the spores are ubiquitous biological agents that are able to infect fruits because of their ability to produce a wide range of hydrolytic enzymes. The growth of fungi depends on many factors such as pH, water activity, temperature, atmosphere, and time [7].

As reported by Amata et al. [8] in Kenya, the total cultivated area of passion fruits was a staggering 10,989 acres yielding 91,091 tons per year. This is worrying because it gives a picture of low fruit growth in Kenya despite their high demand. This explains why although Kenya has a very good climate for fruit growth, it operates suboptimally in fruit production. This has led to high prices of fruits which jeopardizes the health of the country [9].

Due to the demands of pesticide free foods in the consumer market, there is a growing interest in the development of safer, effective alternative compounds to control postharvest fungal diseases [10]. The exploitation of natural products, such as essential oils in controlling plant pathogens, is therefore an idea whose time has come [11].

It is in right of this that the current study was conceived. The study was aimed at isolation and identification of the most prevalent fungal pathogens of passion fruits and carrying out a sensitivity test of the isolates to essential oils of rosemary and eucalyptus which are prevalent in Egerton University and have previously been shown to produce high levels of essential oils [12].

## 2. Materials and Methods

### 2.1. Study Area

The study was conducted at Egerton University, main campus Njoro in Kenya. Egerton University is located in Njoro subcounty with coordinates as 0°23′ south, 35°35′, and altitude of 2000 m above sea level. Temperatures range between 17 and 22°C while the average annual rainfall is 1000 mm [8].

### 2.2. Collection of Infected Passion Fruit Plants

One hundred and fifty samples of infected passion fruits were randomly collected from markets within Egerton University. The samples were weighed before placing in sterile plastic bags, followed by storage in a refrigerator at a temperature of 4 ± 2°C until mycological analysis.

### 2.3. Isolation of Fungal Pathogens

The direct plating technique described by Pitt and Hocking [13] was used. Four small pieces from the margin of lesion of each sample were directly inoculated on prepared plates of potato dextrose agar which contained (g/L) peeled potato 100.0 g, glucose 20.0 g, agar 15.0 g, and water 1000.0 ml after surface sterilization using 70% ethanol for 1 min. The medium was supplemented with chloramphenicol (250 mg per litre) as a bacteriostatic agent [9]. The plates were incubated at 28 ± 1°C for 5 to 7 days. Five replicates were prepared for each sample. The resulting fungi were isolated, purified, and identified according to their macro, micro, and biochemical characteristics using fungal identification keys [14]. Isolation frequency (IF) for each fungus was determined and expressed as percent by using the following formula (Binyam and Girma, 2016):

(1) IF=Number  of  samples  of  occurence  of  fungi  speciesTotal  number  of  samples×100.

### 2.4. Biochemical Characterization of Fungal Pathogens

#### 2.4.1. Amylase Plate Assay

The fungal pathogens were tested for the amylase activity using plate assay [15]. pH of the media was increased to 6.0 using 1 M HCl prior to autoclaving at 121°C for 15 min. Separately, the isolates were inoculated on the medium followed by incubation 28 ± 2°C for 5–7 days. The zone of degradation of starch was determined.

#### 2.4.2. Lipase Plate Assay

Petri dishes containing the medium were separately inoculated with the fungal pathogens and incubated at 28 ± 2°C for 7 days. Appearance of visible precipitate around a fungal colony due to complete degradation of salt of the fatty acid determined lipolytic activity of strains.

#### 2.4.3. Pectinase Plate Assay

Pectinase activity of the isolates was determined according to Ortiz et al. [16]. Culture plates were separately inoculated with fungal pathogens and incubated for 3–5 days at 28 ± 2°C. Following this, the plates were flooded with 1% aqueous hexadecyltrimethyl ammonium bromide.

#### 2.4.4. Protease Plate Assay

Protease activity of the fungal pathogens was determined by inoculating the strains in CZ medium amended with 1% skimmed milk powder [17]. Incubation was carried out in the dark at 28 ± 2°C for 5 days.

### 2.5. Collection of Plant Materials

The plant material used in this study was leaves of rosemary* (Rosmarinus officinalis)* and eucalyptus* (Eucalyptus agglomerata)* growing wild in Egerton University main campus, Njoro. The leaves were placed in sterile polythene paper bags and stored in a deep freezer at 4°C until processing.

### 2.6. Extraction of Essential Oils

A sample of 400 g of fresh rosemary and eucalyptus leaves were separately loaded into 2-litre round bottom flask containing 1.5 litres of water and placed on a heating mantle having power rating of 450 watts and timed. The samples were boiled with water to release the oil within the leaves. The volatile oils evaporated along with the water into the condenser connected to a flask at 100°C and atmospheric pressure. The condensed steam and oils were collected in a separating funnel after which oil and water were separated. The water was drained off gently and the oils were separately collected in a 10 ml measuring cylinder and measured after every 20 min for a period of 3 h. The traces of water in the essential oils were removed by adding 1 gram of magnesium sulphate in the oil as a drying agent after which the yield obtained was calculated using the formula given below [18]:

(2) Yield  of  essential  oil %=amount  of  essential  oil  obtained gamount  of  raw  materials  used g×100.

### 2.7. Determination of Essential Oil Constituents

In order to identify the chemical constituents of the essential oils and therefore determine their quality, the extracted essential oils samples were analyzed using Gas Chromatography Mass Spectrometry (GC-MS) Agilent 6890 gas chromatography instrument coupled to an Agilent 5973 mass spectrometer and an Agilent Chem. The following operating parameters were used for the essential oil sample: capillary GC column HP-5MS 5% phenylmethyl siloxane (30 × 0.25 mm i.d. × 0.25 mm film thickness), a carrier gas helium (flow rate 1.2 mL min^−1^), and a split-less injection mode. Injector temperature was 250°C; oven temperature was set initially at 50°C and then raised to 250°C at 10°C min^−1^ rate till the end of analysis. The eluted analytes were detected using mass selective detector and electron impact ionization (EII) was carried out at 70 eV. The MS fragmentation pattern of the essential oils was compared with those of other essential oils of known composition, with pure compounds and by matching the MS fragmentation patterns with NIST NBS75K mass spectra libraries and those in the literature the essential oils were identified [18].

### 2.8. Sensitivity Test of the Fungal Pathogens to the Essential Oils

Fungi from the pure cultures were separately picked using inoculating needles and put in Bijo bottles that had sterile distilled water in five replicates. Using sterile glass rods the fungi were separately crushed inside the Bijo bottles. Fungus inocula were picked and placed in culture media using a teat pipette. An L shaped glass rod was used to spread the inocula on the culture media. Previously sterilized discs were placed in Bijo bottles having the essential oils. Sterile pairs of forceps were used to put the discs at the centre of the media having the pure cultures of the fungal pathogens. The Petri plates were wrapped using parafilm before incubation at 27°C for 7 days after which the diameter of zones of inhibition was determined.

### 2.9. Data Analysis

Data analysis was carried out using Microsoft excel spreadsheet and Statistical Package for Social Sciences (SPSS) version 17.0 software. Pearson's correlation test was used to determine the relationship between heating period and yield of essential oils while *t*-test was used in comparing yields of essential oils in rosemary and eucalyptus.

## 3. Results

### 3.1. Fungal Pathogens Isolated from Passion Fruits

Cultural and morphological characteristics of the fungal isolates are presented in Table 1. The most prevalent fungal pathogens were* Alternaria *spp. (45%),* Fusarium *spp. (22%), and* Colletotrichum *spp. (17%), and the least was* Penicillium *spp. (16%).

### 3.2. Biochemical Characterization of Fungal Pathogens

The* Alternaria* spp. and* Fusarium* spp. isolated were amylase, pectinase, and protease positive (Table 2). In addition, the isolates tested negative for lipase. Conversely,* Colletotrichum* spp. tested positive for amylase and lipase and negative for pectinase and protease. On the other hand,* Penicillium* spp. were positive for lipase, pectinase, and protease but negative for amylase.

### 3.3. Extraction of Essential Oils

The yield of essential oils varied from 4.5 ± 0.1% after the samples were heated for 180 ± 2 minutes to 0.6 ± 0.3% after heating for 20 ± 2 minutes in rosemary (Table 3). On the other hand, the percentage yield in eucalyptus ranged from 3.5 ± 0.1 after the samples were heated for 180 ± 2 minutes to 0.2 ± 0.2 after heating for 20 ± 2 minutes. The weights of the plant samples, volume of distilled water, and the heating temperature were maintained constant at 400 ± 2 g, 1.5 ± 0.2 L, and 100 ± 2°C, respectively. There was a relationship between heating time and yield of essential oils in rosemary (*r* = 0.99) and leaves (*r* = 0.99). Conversely, there was no significant difference in the amount of essential oils produced by rosemary and eucalyptus (*P* = 0.08).

### 3.4. Constituents of Essential Oils

The percentage composition of myrcene in rosemary was 3.99 ± 0.2 and 5.01 ± 0.3 in eucalyptus, *β*-lin1oo1 (0.45 ± 0.01, 0.50 ± 0.02), nerol (15.00 ± 0.02, 23.98 ± 0.03), citral (18.01 ± 0.02, 14.02 ± 0.02), limonene oxide (0.40 ± 0.03, 1.05 ± 0.02), cineole (9.23 ± 0.03, 10.00 ± 0.01), berbenol (4.56 ± 0.02, 2.22 ± 0.02), and oleic acid (27.67 ± 0.01, 19.98 ± 0.02) (Table 4). There was no significant difference in the percentage composition of the different compounds in the essential oils extract from rosemary and eucalyptus (*P* = 0.47).

### 3.5. Sensitivity of the Fungal Pathogens to Essential Oils

The zone of inhibition of essential oils obtained from rosemary against* Alternaria* spp. was 20 ± 2 mm,* Fusarium* spp. (17 ± 3 mm),* Colletotrichum* spp. (19 ± 3 mm), and* Penicillium* spp. (18 ± 2 mm) (Table 5). On the other hand, the zone of inhibition of eucalyptus essential oils on* Alternaria* spp. was 11 ± 1 mm,* Fusarium* spp. (11 ± 3 mm),* Colletotrichum* spp. (14 ± 2 mm), and* Penicillium* spp. (10 ± 2 mm). There was a significant difference in growth inhibition of the fungal pathogens between essential oils from rosemary and eucalyptus (*P* = 0.000438).

### 3.6. Discussion

*Alternaria* spp. cause diseases such as stem and fruit sports in passion fruits. The findings of this study (Table 1) agree with a previous study in Kenya [8]. The severity of the two diseases is influenced by climatic conditions of a given place which could be a contributing factor. In contrast to the findings of this study, Arielen et al. [15] obtained lower values on isolation of* Fusarium* spp. This difference can be attributed to differences in passion fruit management practices in the two study areas. However, the findings of Dos Santos Silva et al. [19] on isolation of* Colletrotichum* spp. from fruits agree with the findings of the current study. The possible reason could have been the same fruit handling practices during harvesting [20]. In addition, Dinolfo et al. [21] obtained similar results on* Penicillium* spp. as the ones obtained in this study. The possible reason could be similarities in agronomic practices carried out on the plants in the field.

The current study obtained typical biochemical results to the tested fungal pathogens (Table 2). However, the results differed slightly with a previous study carried out in Yemen [3]. Zarali et al. [2] assert that different strains of fungal pathogens exhibit different biochemical characteristics. In addition, mutations due to varying exposure to mutagens could have contributed to the variations in results [19].

El-Shenawy et al. [22] results on isolation of essential oils from rosemary concurred with the ones obtained in this study (Table 3). This may be attributed to the variety from which the essential oils were extracted from being the same. In contrast to the current study on extraction of essential oils from eucalyptus, Seid et al. [18] obtained lower values than those of the current study. This may have arisen from differences in ecological conditions of the two study sites.

However, studies on determination of constituents of essential oils from rosemary carried out in Algeria [12] agreed with those of the current study (Table 4). According to [23] soil mineral composition greatly influences the composition of essential oils in a given plant. In addition, when carrying out a study on antimicrobial activity of essential oils extracted from eucalyptus [24] results disagreed with the findings of the current study. The contributing factor could be differences in extraction procedures [25].

In a previous study carried out in India on sensitivity test of essential oils from rosemary, Macwan et al. [26] obtained results that differ with those of the this study (Table 5). The possible reason could be differences in climatic conditions. However, Sepehri et al. [27] obtained results that supported the findings of this study while carrying out sensitivity test of essential oils from eucalyptus. Similarities of the test pathogens could be a contributing factor [28].

### 3.7. Conclusions and Recommendations

The most prevalent fungal pathogens in maize seeds are* Alternaria* spp.,* Fusarium* spp.,* Colletotrichum* spp., and* Penicillium* spp. Rosemary and eucalyptus spp. produce essential oils that are capable of controlling passion fruits fungal pathogens. There is need to properly handle passion fruit in the farm, during harvesting and transportation, and in the market to avoid fungal pathogens which can lead to fatal human illnesses. In addition, the extraction of essential oils from rosemary and eucalyptus in large scale is highly recommended.

## Figures and Tables

**Table 1:** Common fungal pathogens isolated from passion fruits.

Fungal pathogens	Cultural characteristics	Morphological characteristics	Frequency (%)
*Alternaria *spp.	Large, brown colonies almost filling the whole plate	Septate branched hyphae with brown conidia	45
*Fusarium *spp.	Rapidly growing, wooly to cottonly, lemon shaped, and yellow	Multicellular distinctive sickle shaped macro *conidlia*	22
*Colletotrichum *spp.	Large cottony growth, pink pigmented	Conidia are one-celled, ovoid to oblong, slightly curved at one	17
*Penicillium *spp.	Large fluffy white colonies almost covering the whole surface	Nonseptate branched hyphal enlarged at the apex to form conidiophores; they produce brownish black *conidia *in chains	16

**Table 2:** Biochemical characteristics of the fungal isolates.

Isolate	Amylase	Lipase	Pectinase	Protease
*Alternaria *spp.	+	−	+	+
*Fusarium *spp.	+	−	+	+
*Colletotrichum *spp.	+	+	−	−
*Penicillium *spp.	−	+	+	+

**Table 3:** Yield of essential oils from rosemary and eucalyptus.

Plant	Weight (g)	Distilled H_2_O (L)	Heating time (min)	Temperature (°C)	Yield (%)
Rosemary	400 ± 2	1.5 ± 0.2	20 ± 2	100 ± 2	0.6 ± 0.3
	400 ± 2	1.5 ± 0.2	40 ± 2	100 ± 2	1.0 ± 0.2
	400 ± 2	1.5 ± 0.2	60 ± 2	100 ± 2	1.7 ± 0.2
	400 ± 2	1.5 ± 0.2	80 ± 2	100 ± 2	2.0 ± 0.3
	400 ± 2	1.5 ± 0.2	100 ± 2	100 ± 2	2.8 ± 0.1
	400 ± 2	1.5 ± 0.2	120 ± 2	100 ± 2	3.0 ± 0.3
	400 ± 2	1.5 ± 0.2	140 ± 2	100 ± 2	3.2 ± 0.2
	400 ± 2	1.5 ± 0.2	160 ± 2	100 ± 2	4.1 ± 0.2
	400 ± 2	1.5 ± 0.2	180 ± 2	100 ± 2	4.5 ± 0.1

Eucalyptus	400 ± 2	1.5 ± 0.2	20 ± 2	100 ± 2	0.2 ± 0.2
	400 ± 2	1.5 ± 0.2	40 ± 2	100 ± 2	0.4 ± 0.1
	400 ± 2	1.5 ± 0.2	60 ± 2	100 ± 2	0.6 ± 0.3
	400 ± 2	1.5 ± 0.2	80 ± 2	100 ± 2	1.1 ± 0.3
	400 ± 2	1.5 ± 0.2	100 ± 2	100 ± 2	1.7 ± 9.2
	400 ± 2	1.5 ± 0.2	120 ± 2	100 ± 2	2.0 ± 0.2
	400 ± 2	1.5 ± 0.2	140 ± 2	100 ± 2	2.8 ± 0.1
	400 ± 2	1.5 ± 0.2	160 ± 2	100 ± 2	3.0 ± 0.3
	400 ± 2	1.5 ± 0.2	180 ± 2	100 ± 2	3.5 ± 0.1

**Table 4:** GC-MS analysis of eucalyptus leave oil extracted using different methods.

Compound	Composition (%)	
	Rosemary	Eucalyptus
Myrcene	3.99 ± 0.20	5.01 ± 0.30
*β*-lin1oo1	0.45 ± 0.01	0.50 ± 0.02
Nerol	15.00 ± 0.02	23.98 ± 0.03
Citral	18.01 ± 0.02	14.02 ± 0.02
Limonene oxide	0.40 ± 0.03	1.05 ± 0.02
Cineole	9.23 ± 0.03	10.00 ± 0.01
Berbenol	4.56 ± 0.02	2.22 ± 0.02
Oleic acid	27.67 ± 0.01	19.98 ± 0.02

**Table 5:** Sensitivity of the fungal pathogens to the extracted essential oils.

Fungal pathogen	Zone of inhibition (mm)	
	Essential oils of rosemary	Essential oils of eucalyptus
*Alternaria *spp.	20 ± 2	11 ± 1
*Fusarium *spp.	17 ± 3	11 ± 3
*Colletotrichum*	19 ± 3	14 ± 2
*Penicillium *spp.	18 ± 2	10 ± 2
